# Gait in Huntington’s disease and the stride length-cadence relationship

**DOI:** 10.1186/s12883-014-0161-8

**Published:** 2014-10-01

**Authors:** Mary Danoudis, Robert Iansek

**Affiliations:** Clinical Research Centre for Movement Disorders and Gait, The National Parkinson Foundation Center of Excellence, Kingston Centre, Monash Health, Cheltenham, Victoria 3192 Australia; Monash Ageing Research Centre (MONARC), Monash University, Melbourne, Victoria 3086 Australia; School of Allied Health, College of Sciences, Health and Engineering, Department of Physiotherapy, La Trobe University, Melbourne, Victoria 3086 Australia

**Keywords:** Huntington’s disease, Automatic gait control, Stride length cadence relationship, Intercept, Slope

## Abstract

**Background:**

The progressive deterioration of gait in Huntington’s disease (HD) leads to functional decline and loss of function. To understand the underlying mechanisms responsible for the gait changes in HD, we examined the automatic control of gait by measuring the relationship between stride length and cadence. The relationship is strongly linked in healthy adults during automatic gait but disrupted in pathological gait disorders, such as Parkinson’s disease (PD).

**Methods:**

The stride length cadence relationship was compared between seventeen participants with HD, twenty with PD and twenty one healthy older adults (HOA). Participants had their gait recorded at self-selected preferred, very slow, slow, fast and very fast speeds. Linear regression analysis was used to determine the slope and intercept of the relationship which were compared between groups. The adjustment of stride length and cadence when changing gait speeds was measured and compared within and between groups.

**Results:**

Linearity was strong in all but two participants with HD and one with PD. Slope did not differ between groups (p > 0.05) but intercept was lower in the HD and PD groups compared to HOA (p < 0.05). Stride length was shorter in the HD and PD groups compared to controls at preferred and most adjusted speed conditions (p < 0.05) but cadence did not differ between groups (p > 0.05) regardless of speed. The HD group adjusted stride length and cadence similar to HOA when changing speed. The range of cadence across speed conditions did not differ between groups.

**Conclusion:**

Scaling of stride length but not the regulation of cadence was found to be disrupted in participants with HD.

## Background

Huntington’s disease (HD) is an autosomal dominant inherited neurodegenerative disorder that presents with characteristic gait changes, including decreased walking speed, step initiation difficulties [1-3] and a variable stepping pattern [4-6]. As the disorder progresses mobility worsens, falls risk increases, functional capacity reduces and the need for care increases [7]. There are few studies to support rehabilitation of gait dysfunction in HD [8] partly due to the limited understanding of the underlying mechanisms responsible for the gait changes.

Gait impairments common to HD and PD include hypokinesia and increased gait variability [1,2,9-12]. Disruption to the regulation of step length, greater step to step variability and disturbed gait initiation have been reported to occur before clinical signs of HD appear [2,13] and worsen as disease severity progresses [2,10,13]. Cadence regulation was found to be disrupted in the more advanced stages of HD [2] but remained intact in PD [14]. Variability of angular kinematic gait parameters was shown to be higher in HD and PD compared to controls [9] with chorea possibly contributing to this variability in HD.

The relationship between stride length and cadence (SLCrel) has been used to investigate central gait control mechanisms in healthy adults, pathological gait and lower limb prosthesis users [15-19]. Stride length and cadence are the key determinants of gait speed [17,20]. When healthy adults increase or decrease their self-selected walking speed, they increase or decrease their stride length and cadence in a relatively constant linear relationship [15,17,21]. Once gait speed is set then the relationship between stride length and cadence (SLCrel) functions to maintain it [15,17]. The SLCrel allows for the regulation of gait when minimal attention is required [15,16].

Motor dysfunction in Huntington disease is associated with neuronal loss in the basal ganglia, involving the caudate and putamen nuclei of the striatum and, in more advanced disease, the subthalamic nucleus [22]. Neural networks involving these nuclei are important in the preparation and the execution and maintenance of movement and in executing well learned motor tasks when they shift to being automatic, such as when walking without attention [23]. Disruption to their function results in bradykinesia and akinesia, typical motor deficits seen in HD [22]. The cortical motor areas play a key role in the selection of stride length and the basal ganglia networks are critical in maintaining the selected stride length [24]. Cadence, on the other hand, is thought to be controlled via brainstem connections [24].

The SLCrel has been used to investigate the gait changes in Parkinson’s disease (PD), which also involves dysfunction of the basal ganglia [16]. Parkinson’s disease, like HD, results in a slower gait with shortened steps and normal cadence compared to healthy subjects [25,26]. The slower gait in PD was shown to be due to disruption to the regulation of step length, with cadence control remaining unaffected [16,25,26]. Comparing the SLCrel of these two basal ganglia disorders and comparing them to controls may provide further insights into the functioning of the automatic gait control mechanisms in HD.

This study aimed to investigate the SLCrel in HD. Specifically we aimed to compare the slope and intercept of the SLCrel of participants with HD to that of participants with Parkinson’s disease and healthy controls during self-selected speed walking trials. We proposed the SLCrel would be altered in HD compared to controls and that changes in the relationship would be the same in HD to that found in PD.

## Methods

### Participants

Seventeen people with a diagnosis of HD, based on family history and neurological examination by a neurologist specialized in the disorder, were recruited from a Huntington’s disease data base and from local support groups. Twenty participants with PD were recruited from a Movement Disorders clinic and 21 health older adults (HOA) were recruited from community groups. Inclusion criteria for all participants included the ability to provide informed written consent (a score of ≥24 on the Mini Mental State Examination score, MMSE), being able to walk unassisted over 12 metres and being able to follow instructions. Participants were excluded if they had any other medical condition that interfered with their walking. Ethics approval was obtained from the Southern Health Research Ethics Committee and the Tasmanian Statewide and Mental Health Services Research Committee. All participants provided written informed consent prior to testing.

### Gait assessment

Participants were tested at one of two locations, Melbourne or at the Hobart Menzies Research Institute, Australia. Gait measurements were recorded by a computerized walkway system, the GAITRite® (CIR Systems Inc., USA). The GAITRite® is valid and reliable in measuring gait in people with HD [27], PD [28] and HOA [29]. The electronic walkway used in Melbourne was 8 metres long and that at the Menzies Research Institute 4.6 metres long. To have comparable number of steps recorded by the two systems, gait data was collected over three trials on the longer mat and over five trials on the shorter one for all speed conditions. Gait speed, stride length and cadence were collected at five speeds; preferred, very slow, slow, fast and very fast. The first walking trial for each speed condition was considered to be practice and not used in the analysis. Participants walked at preferred speed first to avoid any influence from the other speed conditions. The remaining walking conditions were counterbalanced to ensure fatigue did not influence the results. Participants commenced walking 2 metres before the start of the mat and finished 2 metres past the end of the mat to avoid acceleration and slowing down during data capture. Participants self-selected their walking speeds in order to minimize attention and maintain ‘automatic’ walking. Standardized instructions, given to each participant prior to the commencement of each walking trial, were to walk at normal/preferred pace and to walk slow, very slow, fast or very fast compared to normal/preferred walking pace. Participants with PD were tested after taking their usual dose of PD medications.

The primary gait variables analyzed were stride length and cadence, with stride length normalized to leg length (mean stride length divided by leg length). The SLCrel was plotted for each participant and was expressed as slope (ratio stride length to cadence as speed changes) and intercept, with data from all speed conditions being used. We reported the intercept at a cadence of 100 steps/min to ensure intercept values were within the data range of all participants [15]. This provided a more valid comparison of the intercept between groups compared to using an intercept of zero cadence. When calculating intercept and slope, cadence was set as cadence-100. A linear model R^2^ value of 0.80 was set as the minimum value for accepting the relationship as linear for SLCrel plots. Based on our previous work, if very fast trials had cadences >150 steps/min and R^2^ values <0.8, these trials were removed and R^2^ values reviewed [15].

Secondary measures investigated the effect of changing speed on normalized stride length (*n*SL) and cadence and included: calculation of the minimum, maximum and range for *n*SL and cadence for each group; determination of the changes to mean *n*SL and cadence in the four adjusted speed conditions compared to the preferred condition for each group; and mean values for normalized gait speed, *n*SL and cadence for all speed conditions compared between groups. Confounders, age and disease duration (for HD and PD groups), were selected as they have been shown to influence gait [30-33]. The effect of psychotropic medications on the SLCrel in the HD group was also investigated.

### Clinical assessment

Participants were characterized according to age, gender, height, leg length, body mass index, MMSE scores and disease duration and severity (HD and PD groups). Psychotropic medications were recorded for the HD group. Disease severity for the HD group was measured using the Unified Huntington’s Disease Rating Scale (UHDRS), motor subscale [34]. The UHDRS has a maximum score of 124, with a higher score indicating greater impairment [34]. Disability was quantified using the Total Functional Capacity (TFC) scale and the Independence Scale [34] [35]. The TFC scale has a maximum score of 13, indicating maximum functional capacity, and is divided into 5 stages; stage 1 indicating early stage and stage 5 indicating late stage [35]. The Independence Scale is scaled in levels from 10 (total bed care) to 100 (asymptomatic). Disease severity for the PD group was rated using the Unified Parkinson’s Disease Rating Scale (UPDRS) motor, maximum score of 56 [36], and the 5 stage Hoehn and Yahr (H&Y) staging tool, with stage 1 indicating mild disease and stage 5 indicating severe disease [37].

### Statistical analysis

Linear regression analysis was used to determine slope, intercept and R^2^ for each participant. One way between groups ANOVA was used to compare group means with Bonferroni post-hoc analyzes. Adjustment was made for age when comparing gait measures, slope, intercept, minimum, maximum and range of *n*SL and cadence values. Adjustment was also made for disease duration when comparing the SLCrel of HD and PD groups. Pearson correlation was computed to analyze relationships between slope and intercept with disease severity for HD and PD groups. Differences in gait speed, stride length and cadence within groups across 5 speed conditions were examined using one way repeated measure ANOVA. Independent-samples-*t*-test was used to compare intercept, slope, and R^2^ in the preferred speed condition for HD participants on or off psychotropic medications. Data was analyzed using SPSS® (v20). Level for statistical significance was set at p ≤ 0.05 [38].

## Results

### Characteristics

Participants in the HD group were younger than PD (p = 0.005) and HOA (p < 0.001) participants (Table 1). MMSE scores were lower in HD (p = 0.03) and PD (p = 0.03) groups compared to controls. Data for CAG repeats, available for 15 participants with HD, ranged from 40 to 51. The mean (SD) total TFC score was 7.9 (3.5). More than 50% of participants were in the early stages of HD; five were in mid and two in late stage. Activity limitation scores for HD group showed 13 required no or minimal assistance with finances and some domestic chores but remained independent with personal care with the remaining four participants living at home but needing assistance with personal care and domestic tasks. Eleven of the HD participants reported taking antipsychotic medications and 13 were prescribed antidepressants.

**Table 1:** **Group characteristics**

	**HD (n = 17)**	**PD (n = 20)**	**HOA (n = 21)**	**p values between groups**
Age, yrs	60.00 (10.5)	68.9 (8.8)	71.7 (4.0)	<0.001
Male, n (%)	9 (53)	16 (80)	8 (38)	-
Height, cm	170.8 (7.2)	171.5 (8.5)	166.6 (6.2)	0.083
Leg length, cm	87.3 (4.7)	89.4 (5.9)	85.6 (3.7)	0.060
BMI	26.9 (7.2)	26.3 (2.4)	24.9 (3.2)	0.362
MMSE	27.4 (2.2)	27.5 (1.9)	29.0 (1.4)	0.011
Disease duration, yrs	7.25 (4.3)	5.6 (5.5)	-	0.430
Disease severity^1^	25.8 (10.63)	13.6 (6.24)	-	n/a
Disease stage^2^	2 (IQR 1,3)	2 (IQR 1,3)	-	n/a

Note. Means and SD unless stated otherwise; *HD* Huntington’s disease, *PD* Parkinson’s disease, *HOA* healthy older adults; *BMI* body mass index, *MMSE* Mini Mental State Examination, *IQR* inter quartile range; Disease severity, ^1^Unified Huntington’s Disease Rating Scale total motor score for HD group and Unified Parkinson’s Disease Rating Scale total motor score part III for the PD group; Disease stage, ^2^Stage of disease based on Total Functional Capacity scores for HD group, Hoehn & Yahr for PD group; n/a, not applicable.

All but one participant in the PD group were taking Parkinson’s medications. Time from when PD medications were taken to when testing commenced ranged from within 30 minutes up to 4.5 hours, average time being 1.9 hours. Dyskinesia was not present in the PD participants. Disease severity was mild, with 13 participants at Hoehn and Yahr early stages 1–2 and five at mid stage 3. Motor impairment was correspondingly mild with 14 having a UPDRS motor score of 15/56 or less.

### Gait measures

At preferred speed, HD and PD groups walked slower compared to HOA (p <0.05) due to reduction of *n*SL (p < 0.05) (Table 2). After adjusting for age results for speed and *n*SL were unchanged however cadence was lower in the HD group compared to PD (p = 0.004) and HOA groups (p < 0.001).

**Table 2:** **Gait parameters for adjusted speed conditions compared between groups**

**Parameter**	**HD v HOA**	**HD v PD**	**PD v HOA**
*n*speed very slow	0.68 (0.34) v 0.86 (0.22) p = 0.11	0.68 (0.34) v 0.82 (0.22) p = 0.35	0.82 (0.22) v 0.86 (0.22) p = 1.0
*n*speed slow	1.01 (0.36) v 1.16 (0.20) p = 0.17	1.00 (0.36) v 1.09 (0.18) p = 0.95	1.09 (0.18) v 1.16 (0.20) p = 1.0
*N*speed preferred	1.30 (0.35) v 1.52 (0.17) p = 0.02	1.31 (0.32) v 1.31 (0.21) p = 1.0	1.31 (0.21) v 1.52 (0.17) p = 0.02
*n*speed fast	1.65 (0.42) v 1.86 (0.18) p = 0.08	1.65 (0.42) v 1.53 (0.21) p = 0.69	1.53 (0.21) v 1.86 (0.18) p = 0.002
*n*speed very fast	2.07 (0.44) v 2.29 (2.32) p = 0.12	2.07 (0.44) v 1.92 (0.28) p = 0.48	1.92 (0.28) v 2.29 (2.32) p = 0.001
*n*SL very slow	1.00 (0.33) v 1.29 (0.15) p = 0.001	1.00 (0.33) v 1.15 (0.23) p = 0.19	1.15 (0.23) v 1.29 (0.15) p = 0.20
*n*SL slow	1.25 (0.30) v 1.46 (0.12) p = 0.01	1.25 (0.30) v 1.32 (0.18) p = 0.89	1.32 (0.18) v 1.46 (0.12) p = 0.09
*n*SL pref	1.44 (0.28) v 1.63 (0.10) p = 0.02	1.44 (0.28) v 1.43 (0.20) p = 1.0	1.43 (0.20) v 1.63 (0.10) p = 0.01
*n*SL fast	1.60 (0.26) v 1.78 (0.12) p = 0.02	1.60 (0.26) v 1.55 (0.20) p = 1.0	1.55 (0.20) v 1.78 (0.12) p = 0.001
*n*SL very fast	1.74 (0.25) v 1.91 (0.18) p = 0.08	1.74 (0.25) v 1.69 (0.23) p = 1.0	1.69 (0.23) v 1.91 (0.18) p = 0.01
Cadence very slow	78.89 (17.47) v 79.14 (14.0) p = 1.0	78.89 (17.47) v 84.39 (11.73) p = 0.76	84.39 (11.73) v 79.14 (14.00) p = 0.75
Cadence slow	94.64 (16.81) v 95.32 (10.92) p =1.0	94.64 (16.81) v 99.47 (9.83) p = 0.75	99.47 (9.83) v 95.32 (10.92) p = 0.89
Cadence pref	106.1 (14.46) v 112.03 (9.17) p = 0.26	106.1 (14.46) v 110.22 (6.96) p = 0.70	110.22 (6.96) v 112.03 (9.17) p = 1.0
Cadence fast	121.9 (17.58) v 124.8 (10.17) p =1.0	121.9 (17.58) v 118.9 (6.28) p = 1.0	118.9 (6.28) v 124.8 (10.17) p = 0.35
Cadence very fast	141.7 (16.33) v 144.8 (12.69) p =1.0	141.7 (16.33) v 136.1 (11.76) p = 0.65	136.1 (11.76) v 144.8 (12.69) p = 0.14

Note. Means and SD unless stated otherwise; *HD* Huntington’s disease, *HOA* healthy older adults, *PD* Parkinson’s disease; *n*speed, normalized speed, *n*SL, normalized stride length.

The HD group had shorter *n*SL compared to controls in most adjusted speed conditions (Table 2). Cadence did not differ between groups for all speed conditions (p > 0.05). Adjusting for age resulted in *n*speed being significantly slower in the HD group compared to the PD and control groups at very slow speed (p = 0.02, p = 0.003 respectively) and slower than controls at slow gait speed (p = 0.015). Adjusting for age did not change results for *n*SL however cadence in the HD group was lower than controls in the fast speed condition (p = 0.017) and lower in the PD group compared to controls at very fast speed (p = 0.03).

### Stride length cadence relationship - SLCrel

Inspection of the SLCrel plots in the HD group showed nonlinear relationships for two participants, with R^2^ values 0.67 and <0.001 and no trials with a cadence >150 steps/min. Their data was not included in the slope and intercept analysis. All other relationships were positive and linear with R^2^ values ≥ 0.82. One participant in the PD group, excluded from the SLCrel analysis, had a R^2^ value of 0.39. The remaining PD participants had R^2^ values ≥0.85. One control participant had a R^2^ value 0.66, which, after removal of trials with cadences >150 steps/min, was R^2^ = 0.9. All remaining HOA participants had R^2^ ≥ 0.82.

### Intercept and slope

**Figure 1:**
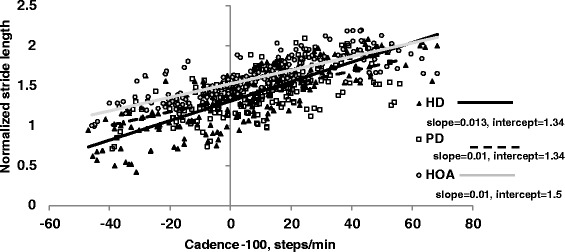
**SLCrel for 3 groups, cadence set at cadence-100.** NOTE SLCrel, stride length-cadence relationship.

Figure 1 shows *n*SL plotted against cadence including all data points for all participants and mean regression lines for each group. The HD and PD groups had lower intercepts compared to the HOA group (p≤0.05) but slope did not differ between groups (p = 0.26) (Table 3). Adjusting for age did not change results for intercept, (p = 0.009), or slope (p = 0.24). Intercept and slope did not differ between the HD and PD groups (p=1.0, p=0.43 respectively) and results did not change after adjusting for disease duration (p = 0.88).

**Table 3:** **SLCrel and minimum, maximum and range of nSL, cadence**

	**HD (n = 15)**	**PD (n = 19)**	**HOA (n = 21)**	**p values between groups**
Slope	0.013 (0.004)	0.011 (0.004)	0.011 (0.005)	0.259
Intercept	1.34 (0.208)	1.34 (0.210)	1.49 (0.101)	0.012
R^2^	0.90 (0.047)	0.94 (0.054)	0.95 (0.043)	0.011
Min nSl	0.940 (0.322)	1.124 (0.233)	1.271 (0.146)	0.001
Max nSL	1.791 (0.265)	1.721 (0.232)	1.931 (0.168)	0.013
Range nSl	0.853 (0.300)	0.597 (0.244)	0.660 (0.179)	0.009
Min cad	73.03 (14.09)	82.34 (11.67)	77.70 (13.94)	0.135
Max cad	140.87 (14.78)	137.08 (12.26)	144.71 (12.14)	0.187
Range cad	67.8 (20.27)	54.7 (15.62)	67.01 (20.16)	0.069

Note. Non adjusted results; Means and SD unless stated otherwise; *HD* Huntington’s disease, *PD* Parkinson’s disease, *HOA* healthy older adults, *R* ^*2*^ coefficient of determination, *Min nSL* minimum normalized stride length, *Max nSL* maximum normalized stride length, *Min cad* minimum cadence, *Max cad* maximum cadence.

In the HD group, intercept correlated with disease severity, UHDRS scores, *r* = −.81, p < .001; slope had a medium but not significant relationship with disease severity, *r* = .47, p = 0.08; and there was a significant correlation between R^2^ and disease severity, *r* = −.52, p = 0.05. Results were similar for the PD group, with intercept but not slope or R^2^ correlating with disease severity, UPDRS scores, *r* = −.67, p = 0.002, *r* = −.009, p = 0.97 r = -.39, p = .095 respectively. Comparison between HD participants on or not on antipsychotic medications found no difference for intercept, slope or R^2^ values (p > 0.05).

### Modulation of gait speed, stride length and cadence

Participants in the HD group did not differ to controls or to the PD group for their mean minimum and maximum or range of cadence across speed conditions (p > 0.05) (Table 3). The HD group did however have a smaller minimal *n*SL (p < 0.001) and shorter but not significant maximum *n*SL (p = 0.20) compared to controls (Table 3). The mean minimum *n*SL of the PD group did not differ to the HD or control groups (p>0.05) however their mean maximum *n*SL was shorter than controls (p = 0.01). There was a trend for the HD group’s range for *n*SL to be greater than that of HOA (p = 0.06) but was greater compared to the PD group (p = 0.009) (Table 3). Adjusting for age did not change these results.

**Figure 2:**
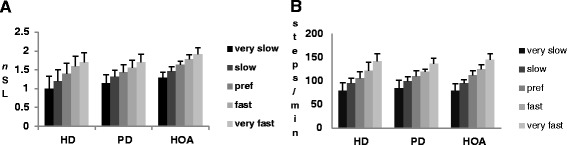
**Comparison of mean normalized stride length (A) and cadence (B) values across 5 speed conditions for HD, PD and HOA groups.** All groups had significant change in *n*stride and cadence in four speed conditions compared to their preferred condition (p < 0.001).

All groups decreased or increased their speed significantly compared to their preferred speed (p < 0.05) with a corresponding significant increase or decrease of *n*SL and cadence for each speed condition compared to baseline (p < 0.05) (Figure 2). The HD group adjusted their *n*SL and cadence when walking slower or faster than their preferred speed similar to controls. For example the percentage change of *n*SL in the adjusted speed conditions compared to preferred *n*SL, HD versus controls was: very slow speed −30.6% versus −20.9%; slow speed −13.2% versus 10.4%; fast speed 11.1% versus 9.42%; and very fast speed 20.8% versus 17.2%. The PD group also made similar adjustments to *n*SL and cadence compared to controls.

## Discussion

This is the first study to investigate the SLCrel in HD. The key finding was the lower intercept for the HD group compared to controls. In other words, for any given cadence, stride length was reduced in HD compared to controls, consistent with a reduced scaling capacity. All except two participants with HD and one participant with PD demonstrated a strong linear relationship between stride length and cadence. Participants with HD were able to modulate their walking speed by adjusting both stride length and cadence in the same proportion to the HOA and PD groups.

The HD group had a lower intercept of their SLCrel compared to controls suggesting their stride length is scaled lower regardless of gait speed. The PD group also demonstrated a lower intercept, which has been previously reported [16,18]. The reduction in intercept did not affect slope; the stride length cadence ratio for the HD and PD groups did not differ to controls. The lower intercept found in PD participants has been shown to increase toward that of controls after taking levodopa [16]. Levodopa improves stride length in PD by its action on the basal ganglia. This suggests similar pathways in the basal ganglia may be disrupted in both HD and PD and that gait speed is reduced in both groups due to disruption to the regulation of stride length by the basal ganglia. Intercept was negatively associated with disease severity in both groups which is consistent with step length shortening in both HD and PD as these disorders progress [2,39].

Studies of healthy adults have demonstrated that the ratio between stride length and cadence, as measured by slope of the SLCrel plot, is consistent as speed changes except possibly at higher speeds [15,17,40]. The consistency of the SLCrel slope has also been demonstrated in PD and Progressive Supranuclear palsy [16,18]. This current study also showed the HD group, when adjusting their walking speed, modulated stride length and cadence in a constant ratio with slope not differing to that of the PD and control groups. Walking at self-selected speed in a stable environment with minimal attention is controlled by the basal ganglia through its connections to frontal cortical regions [41]. We suggest that these neural connections are responsible for maintaining the stable relationship between stride length and cadence which in turn allows for the running of self-selected speed in automatic mode. The findings also suggest the central pathways responsible for this regulation were intact in this sample. Slope was not associated with disease severity however this result may be influenced by the mild to moderate disease severity of participants included in this sample. Future studies need to investigate the SLCrel slope in participants with more severe disease.

People with HD were able increase or decrease their walking speed by making similar adjustments to controls of their stride length and cadence, which is agreement with past studies [3-5]. We showed both stride length and cadence increased as speed increased however past studies reported variable contributions to changing gait speed [3,4]. We also demonstrated that cadence did not differ between HD and HOA groups across all speed conditions nor did their minimum and maximum cadences and range of change in cadence differ, which has not been previously reported. These findings suggest timing of gait may be intact in HD.

The use of strategies shown to normalize stride length in PD [39] have not been investigated in HD. The difficulty in synchronizing stepping to external cueing devices in HD is reported to be due to attentional deficits [6] limiting their clinical usefulness in gait rehabilitation for HD with impaired cognition. In addition the use of a metronome aims to increase gait speed by increasing cadence and does not address disruption to step length regulation. Future research is required to evaluate the effectiveness of specific interventions, in particular strategies to increase step length using visual cues or attention [39], on improving gait in HD.

There were limitations to this study. Most participants in the HD and PD groups were in the early stages of the disorders and may not be representative of those with more severe disease. This sample of participants with HD was heterogeneous however it was beyond the scope of this study to recruit a larger sample because of the difficulty in identifying people who met the inclusion criteria. The resulting small numbers in each of the four stages of the disorder meant comparison of gait and the SLCrel between the HD subgroups could not be done. The small numbers also meant this study could not investigate whether the SLCrel of HD participants who were predominantly hypokinetic differed from those participants with significant chorea. The inclusion of only two participants in the HD group with marked disruption to the SLCrel prevented any meaningful investigation of the relationship between disrupted SLCrel and disease severity and disease duration. Future studies need to investigate the SLCrel across the HD stages with larger numbers to determine if this relationship remains stable over the course of the disorder. The HD group also demonstrated a greater range of *n*SL compared to the PD group and control group. The basis for this finding is unclear but may reflect the tendency in HD for chorea however this was not explored in this current study. This is a possible topic for future research. As measures of disease severity differed for the HD and PD groups, it was not possible to determine if disease severity contributed to differences between them. Levodopa has been shown to increase the intercept in PD [16]. In this current study participants with PD were only tested after taking levodopa. Future studies need to compare the SLCrel of PD participants before levodopa medication with that of HD participants.

## Conclusion

This study provides further insights into the mechanisms contributing to the gait changes in HD. These results will provide guidance to clinicians in the development of effective interventions to improve mobility and function in people with HD.

## Abbreviations

HD: Huntington’s disease
PD: Parkinson’s disease
HOA: healthy older adults
SLCrel: Stride length cadence relationship
MMSE: Mini mental state examination
*n*SL: Normalized stride length
UHDRS: Unified Huntington’s Disease Rating Scale
TFC: Total Functional Capacity
UPDRS: Unified Parkinson’s Disease Rating Scale
H&Y: Hoehn and Yahr staging tool

## References

[1] Koller WC, Trimble J (1985). The gait abnormality of Huntington’s disease. Neurology.

[2] Rao AK, Muratori L, Louis ED, Moskowitz CB, Marder KS (2008). Spectrum of gait impairments in presymptomatic and symptomatic Huntington’s disease. Mov Disord.

[3] Thaut MH, Miltner R, Lange HW, Hurt CP, Hoemberg V (1999). Velocity modulation and rhythmic synchronization of gait in Huntington’s disease. Mov Disord.

[4] Bilney B, Morris ME, Churchyard A, Chiu E, Georgiou-Karistianis N (2005). Evidence for a disorder of locomotor timing in Huntington’s disease. Mov Disord.

[5] Churchyard AJ, Morris ME, Georgiou N, Chiu E, Cooper R, Iansek R (2001). Gait dysfunction in Huntington’s disease: parkinsonism and a disorder of timing. Implications for movement rehabilitation. Adv Neurol.

[6] Delval A, Krystkowiak P, Delliaux M, Blatt J-L, Derambure P, Destée A, Defebvre L (2008). Effect of external cueing on gait in Huntington’s disease. Mov Disord.

[7] Wheelock VL, Tempkin T, Marder K, Nance M, Myers RH, Zhao H, Kayson E, Orme C, Shoulson I, Huntington Study Group (2003). Predictors of nursing home placement in Huntington disease. Neurology.

[8] Busse ME, Rosser AE (2007). Can directed activity improve mobility in Huntington’s disease?. Brain Res Bull.

[9] Delval A, Krystkowiak P, Blatt JL, Labyt E, Dujardin K, Destee A, Derambure P, Defebvre L (2006). Role of hypokinesia and bradykinesia in gait disturbances in Huntington’s disease: a biomechanical study. J Neurol.

[10] Hausdorff JM, Cudkowicz ME, Firtion R, Wei JY, Goldberger AL (1998). Gait variability and basal ganglia disorders: stride-to-stride variations of gait cycle timing in parkinson’s disease and Huntington’s disease. Mov Disord.

[11] Reynolds NC, Myklebust JB, Prieto TE, Myklebust BM (1999). Analysis of gait abnormalities in Huntington disease. Arch Phys Med Rehabil.

[12] Morris ME, Iansek R, Matyas TA, Summers JJ (1994). The pathogenesis of gait hypokinesia in Parkinson’s disease. Brain.

[13] Delval A, Bleuse S, Simonin C, Delliaux M, Rolland B, Destee A, Defebvre L, Krystkowiak P, Dujardin K (2011). Are gait initiation parameters early markers of Huntington’s disease in pre-manifest mutation carriers?. Gait Posture.

[14] Morris ME, Iansek R, Matyas TA, Summers JJ (1994). Ability to modulate walking cadence remains intact in Parkinson’s disease. J Neurol Neurosurg Psychiatry.

[15] Egerton T, Danoudis M, Huxham F, Iansek R (2011). Central gait control mechanisms and the stride length cadence relationship. Gait Posture.

[16] Morris M, Iansek R, Matyas T, Summers J (1998). Abnormalities in the stride length-cadence relation in parkinsonian gait. Mov Disord.

[17] Zijlstra W, Rutgers AWF, Hof AL, Van Weerden TW (1995). Voluntary and involuntary adaptation of walking to temporal and spatial constraints. Gait Posture.

[18] Egerton T, Williams D, Iansek R (2012). Comparison of gait in progressive supranuclear palsy, Parkinson’s disease and healthy older adults. BMC Neurol.

[19] Howard C, Wallace C, Stokic DS (2013). Stride length-cadence relationship is disrupted in below-knee prosthesis users. Gait Posture.

[20] Grieve DW, Gear RJ (1966). The relationships between length of stride, step frequency, time of swing and speed of walking for children and adults. Ergonomics.

[21] Du Chatinier K, Molen NH, Rozendal RH (1970). Step length, step frequency and temporal factors of the stride in normal human walking. Proceed Koninklijke Nederlandse Akademie van Wetenschappen Series C Biol Med Sci.

[22] Guo Z, Rudow G, Pletnikova O, Codispoti KE, Orr BA, Crain BJ, Duan W, Margolis RL, Rosenblatt A, Ross CA, Troncoso JC (2012). Striatal neuronal loss correlates with clinical motor impairment in Huntington’s disease. Mov Disord.

[23] Wu T, Wang J, Wang C, Hallett M, Zang Y, Wu X, Chan P (2012). Basal ganglia circuits changes in Parkinson’s disease patients. Neurosci Lett.

[24] Snijders AH, Leunissen I, Bakker M, Overeem S, Helmich RC, Bloem BR, Toni I (2011). Gait-related cerebral alterations in patients with Parkinson’s disease with freezing of gait. Brain.

[25] Ferrandez AM, Blin O (1991). A comparison between the effect of intentional modulations and the action of L-dopa on gait in Parkinson’s disease. Beh Brain Res.

[26] Morris ME, Iansek R, Matyas TA, Summers JJ (1996). Stride length regulation in Parkinson’s disease. Normalization strategies and underlying mechanisms. Brain.

[27] Rao AK, Quinn L, Marder KS (2005). Reliability of spatiotemporal gait outcome measures in Huntington’s disease. Mov Disord.

[28] Nelson AJ, Zwick D, Brody S, Doran C, Pulver L, Rooz G, Sadownick M, Nelson R, Rothman J (2002). The validity of the GaitRite and the functional ambulation performance scoring system in the analysis of Parkinson gait. NeuroRehabilitation.

[29] Menz HB, Latt MD, Tiedemann A, Mun San Kwan M, Lord SR (2004). Reliability of the GAITRite walkway system for the quantification of temporo-spatial parameters of gait in young and older people. Gait Posture.

[30] Nemanich ST, Duncan RP, Dibble LE, Cavanaugh JT, Ellis TD, Ford MP, Foreman KB, Earhart GM (2013). Predictors of gait speeds and the relationship of gait speeds to falls in men and women with Parkinson disease. Parkinson’s Dis.

[31] Schrag A, Ben-Shlomo Y, Quinn N (2002). How common are complications of Parkinson’s disease?. J Neurol.

[32] Mahant N, McCusker EA, Byth K, Graham S (2003). Huntington’s disease: clinical correlates of disability and progression. Neurology.

[33] Marder K, Zhao H, Myers RH, Cudkowicz M, Kayson E, Kieburtz K, Orme C, Paulsen J, Penney JB, Siemers E, Shoulson I (2000). Rate of functional decline in Huntington’s disease. Huntington study group. Neurology.

[34] Huntington Study Group (1996). Unified Huntington’s disease rating scale: reliability and consistency. Mov Disord.

[35] Shoulson I, Fahn S (1979). Huntington disease: clinical care and evaluation. Neurology.

[36] Fahn S, Elton RL, Fahn S (1987). UPDRS Development Committee. Unified Parkinson’s Disease Rating Scale. Recent Developments in Parkinson's Disease.

[37] Hoehn MM, Yahr MD (1967). Parkinsonism: onset, progression and mortality. Neurology.

[38] Perneger TV (1998). What's wrong with bonferroni adjustments. BMJ.

[39] Morris ME (2000). Movement disorders in people with Parkinson disease: a model for physical therapy. Phys Ther.

[40] Nilsson J, Thorstensson A, Halbertsma J (1985). Changes in leg movements and muscle activity with speed of locomotion and mode of progression in humans. Acta Physiol Scand.

[41] Wu T, Chan P, Hallett M (2010). Effective connectivity of neural networks in automatic movements in Parkinson's disease. NeuroImage.

